# Primary care physician supply and other key determinants of health care utilisation: the case of Switzerland

**DOI:** 10.1186/1472-6963-8-8

**Published:** 2008-01-11

**Authors:** André Busato, Beat Künzi

**Affiliations:** 1 Institute for Evaluative Research in Orthopaedic Surgery, University of Bern, Stauffacherstrasse 78, CH-3014, Bern, Switzerland; 2 Swisspep – Institute for Quality and Research in Healthcare, Postfach – CH 3073 Guemligen, Switzerland

## Abstract

**Background:**

The Swiss government decided to freeze new accreditations for physicians in private practice in Switzerland based on the assumption that demand-induced health care spending may be cut by limiting care offers. This legislation initiated an ongoing controversial public debate in Switzerland. The aim of this study is therefore the determination of socio-demographic and health system-related factors of per capita consultation rates with primary care physicians in the multicultural population of Switzerland.

**Methods:**

The data were derived from the complete claims data of Swiss health insurers for 2004 and included 21.4 million consultations provided by 6564 Swiss primary care physicians on a fee-for-service basis. Socio-demographic data were obtained from the Swiss Federal Statistical Office. Utilisation-based health service areas were created and were used as observational units for statistical procedures. Multivariate and hierarchical models were applied to analyze the data.

**Results:**

Models within the study allowed the definition of 1018 primary care service areas with a median population of 3754 and an average per capita consultation rate of 2.95 per year. Statistical models yielded significant effects for various geographical, socio-demographic and cultural factors. The regional density of physicians in independent practice was also significantly associated with annual consultation rates and indicated an associated increase 0.10 for each additional primary care physician in a population of 10,000 inhabitants. Considerable differences across Swiss language regions were observed with reference to the supply of ambulatory health resources provided either by primary care physicians, specialists, or hospital-based ambulatory care.

**Conclusion:**

The study documents a large small-area variation in utilisation and provision of health care resources in Switzerland. Effects of physician density appeared to be strongly related to Swiss language regions and may be rooted in the different cultural backgrounds of the served populations.

## Background

Previous research on variations in the volume of physician services in geographic areas has emphasized that an additional use of services is not necessarily associated with improved health in the corresponding populations[1]. After controlling for input prices and health status, it was found that the volume of physician services is driven partly by local practice patterns and partly by differences in physician density and speciality. However, an association between greater volume and demonstrable improvement in outcomes was not found [2].

Such findings may have contributed to a governmental decision to freeze new accreditations for physicians in private practice in Switzerland[3]. This legislation is based on the assumption that demand-induced health care spending may be cut by limiting the associated care offers, a policy that initiated a controversial public debate in Switzerland. Therefore, the purpose of this article is to provide estimates of resource utilisation in Swiss primary care and to determine relevant factors in this context.

The Swiss health system is based on principles of free demand and supply and most services are reimbursed on a fee-for-service system. The system provides comprehensive coverage of high quality services, however, the financial costs rank second (after the US) among OECD countries and are still rising. Swiss health care is characterized by federalist structures and is fragmented into 26 cantonal health systems[4,5]. National authorities have little responsibility and nationwide reforms aimed at cutting health care expenditures are therefore difficult to achieve.

Due to this highly decentralized governing of Swiss health care, the specific goals of the study include the estimation of spatial variability of availability and utilisation of primary care resources, taking into account demographic, socioeconomic, and cultural attributes of the population.

One of the problems associated with an estimation of the regional availability of health care is that patients frequently travel outside their residential areas to seek care, especially those residing in remote and non-urban areas. The formation of meaningful health service areas as a unit of analysis is thus an important issue as it allows the control of demand-induced utilisation phenomena, including medical consumerism. We have defined health care service areas for Swiss primary care based on the complete claims data of compulsory health insurance obtained from the data pool of all Swiss health insurers (santesuisse) and used these areas as the units of analysis. We therefore applied a new and alternative spatial model to describe the utilisation and provision of primary health care in Switzerland.

## Methods

1018 ambulatory care service areas were created according to Goodman* et al.*[6] based on 2761 communities and the community area codes of primary care physician practices and the residential addresses of their patients. For each service area, socio-demographic attributes were calculated using community data available from the Swiss Federal Statistical Office. The localisation index (LI) and the market share index (MSI) were calculated as measures of the health care utilisation and provision in each area. The LI indicates the degree of localization of primary care provided for the population in a given area and the MSI indicates the degree of localization of primary care provision from the provider's perspective [7].

### Data

Complete frequency data of all consultations reimbursed by the compulsory basic health insurance were obtained from the data pool of all Swiss health insurers (santésuisse) for all Swiss ambulatory care physicians for the year 2004. These data consisted of two files. The first file included a list of all Swiss ambulatory care providers classified into 49 different medical specialties with board certifications according to the Swiss Medical Association (FMH), together with the area codes of the practice locations. For this study, we used only consultations provided by primary care physicians (FMH board certifications for primary care, general internal medicine, general practitioner without board certification, incorporated group practice member). The second file included consultation frequencies of each physician classified by gender, by 20 age groups, and by community of patient residence. Patients' communities were therefore the smallest observational unit of this study. We used only data from consultations provided for illness and maternity. Accident-related consultations were excluded due to different health insurance coverage.

### Data analysis

Descriptive analyses included the calculation of means and medians, depending on the distribution of the data. Univariate associations between variables were assessed with Spearman correlation coefficients (rho). Statistical models had to account for the two-level hierarchical structure of the data; i.e. level I data at the level of 2762 community levels and level II data at the level of 1018 service areas; consequently, multilevel models were used[8].

The main outcome variable of these models was the annual per capita consultation rate with a primary care physician for each community in the year 2004. Preliminary analyses indicated a symmetrical and almost normal distribution of this outcome variable. The following explanatory variables were analysed:

#### Level I (community attributes)

- Average age of the population

- Female-to-male ratio

- Language code of the community (German, French, Italian, Romansh)

- Proportion of non-Swiss citizens among the residents in each community

- A nine level classification of communities: Urban, suburban, high income, peri-urban, tourist, industrial-tertiary, rural-commuter, agricultural-mixed, agricultural rural. This typology was developed by the Swiss Federal Statistical Office in order to provide a meaningful representation of communal characteristics for demographic and socioeocomic investigations [9]. The classification is mainly based on the principle of hierarchic relationships between urban centres and peripheral communities.

#### Level II (service area attributes)

- Area code of the service area (clustering variable)

- Number of primary care physicians per 10'000 inhabitants (GP density)

- Number of other, non-primary care physicians per 10'000 inhabitants (specialist density) in the same service area

- Presence of a hospital providing ambulatory services in the same service area (coded as a binary variable with 0 for no hospital and 1 for at least one hospital).

Additionally, random effects variables for the intercept, average age, and female-to-male ratio were included in the model. The decision to include these random effects variables was based on preliminary analyses of heterogeneity of intercepts and slopes. The structure of the variance-covariance matrix was specified as unstructured. Other level I variables such as the income distribution within communities and the ratio of > 65 vs. < 20 years of age also were analysed initially. However, based on the extent of the respective variance components and the criteria of model fit, the set of level I variables mentioned above appeared to account for most of the outcome variance and provided the best fit between observed and expected data. A further analysis indicated significant first-order interactions between GP density and language region, between non-Swiss population and language region, and finally between female-to-male ratio and non-Swiss among the resident population. The corresponding interaction terms were thus also included in the model. Consequently, the final model used in this study had the following structure (fixed effects are denoted with *γ*_*ij*_, random effects with *μ*_*ij *_and the redidual error is denoted with *r*_*ij*_):

*Y*_*ij *_= *γ*_*00 *_+ *γ*_*01 *_*GP density*_*j *_+ *γ*_*02 *_*specialist density*_*j *_+ *γ*_*03 *_*hospital*_*j *_+ *γ*_*10 *_*age*_*i *_+ *γ*_*20 *_*FM-ratio*_*i *_+ *γ*_*30 *_*non-Swiss*_*i *_+ *γ*_*40 *_*language*_*i *_+ *γ*_*50 *_*community-type*_*i *_+ *γ*_*11 *_*GP density*_j _*language_*i *_+ *γ*_*60 *_*non-Swiss*_i _*language_*i *_+ *γ*_*70 *_*FM-ratio*_i _*non-Swiss_*i *_+ *μ*_*0j *_+ *μ*_*1j *_*age*_*i *_+ *μ*_*2j *_*MF-ratio*_*i *_+ *r*_*ij*_

Additional language-stratified analyses were performed because of significant language-related first-order interaction terms. Since there are only a few communities in which Romansh is spoken, stratified analyses were restricted to German-, French-, and Italian-speaking communities only. The stratified models comprised the same set of explanatory variables except language region and the related interaction terms. Presence of hospital, language region, and community type were treated as classification variables, and continuous explanatory variables were centred to facilitate parameter interpretation. Results for classified data were interpreted as least-square means (LS-Means) with 95% confidence intervals (CI95), and the Bonferroni procedure was used to adjust for multiple comparisons. Variance components of level II variables were additionally calculated[10]. Variance components quantify the corresponding proportions of outcome variation accounted for by specific variables and therefore provide estimates about the relevance of regional variables within Swiss primary care. Model fit was assessed using residual analyses and the Akaike Information Criterion (AIC) was used to compare several versions of the model during model development. Residual analysis showed no violation of basic assumption for linear models. SAS 9.1 (SAS Institute Inc., Cary, NC, USA) and "proc mixed" were used for all analyses and the level of significance was set at 0.05 throughout the study.

## Results

The data obtained from the pool of Swiss health insurers (santésuisse) included the complete records of the 21'413'299 consultations of all 6564 Swiss primary care physicians with patients throughout Switzerland that were reimbursed by compulsory health insurance in 2004. These primary care data accounted for 39.5% of all ambulatory care physicians (i.e. primary care physicians and specialists) and 56.5% of all ambulatory consultations reimbursed by basic health insurance (consultations provided by ambulatory departments of hospitals were not included). The geographic distribution of physicians and patient consultations across different community types is given in Table 1.

**Table 1:** Distribution of physicians and consultation frequencies across community types (2760 communities)

Type of community^a^	Number/proportion of physicians	Physicians per 10,000 inhabitants	Proportion of consultations	Per capita consultation rate
Urban centres	2703 (41.2%)	9.3	29.7%	3.04
Suburban	1694 (25.8%)	7.9	28.9%	2.86
High income	311 (4.7%)	9.6	3.9%	2.23
Periurban	470 (7.2%)	7.8	10.0%	2.62
Tourist	235 (3.6%)	9.6	3.6%	2.98
Industrial-tertiary	655 (9.9%)	8.0	11.1%	3.18
Rural-commuter	218 (3.3%)	7.5	5.9%	2.57
Agricultural-mixed	246 (3.8%)	7.7	5.8%	2.63
Agricultural rural	32 (0.6%)	8.7	1.0%	2.64

Overall	6564 (100%)	8.0	100%	2.95

^a ^Classification according to the Swiss Federal Statistical Office

Ambulatory care service areas were constructed from community codes of physicians and patients[6,11] without setting constraints on either population size or localisation of utilizing or providing health services. 1018 service areas emerged with at least one physician per area. Service area-specific data on demographic and socioeconomic attributes, and health care utilisation were calculated based on the associated statistics of the included communities (Table 2). These data indicate a wide range in how primary care is provided and consumed across various regions. Population data show a few urban areas with very large populations that skew the corresponding mean to the right. However, 75% of all service areas had less than 6750 inhabitants. Similar observations were also made for the overall number of primary care physicians within the areas (Table 2). 75% of all the areas had fewer than 5 physicians. Less skewed, but still highly variable, data were observed for the measures of utilisation and provision of primary care in terms of the consultation frequencies LI and MSI. The most important variable in this context was the annual per capita consultation rate with a primary care physician, which showed an eleven-fold variation across the regions. Consequently, this variable was selected as the main outcome of the statistical procedures. Positive and significant correlations were found between regional population size and both LI (rho = 0.44) and MSI (rho = 0.48), implying a higher localisation of utilisation and provision of resources in highly populated areas.

**Table 2:** Attributes of 1018 primary care service areas

	Average	Median	Min	Max
No. of communities	2.71	2	1	61
Population size	7318	3754	282	419599
Average age	38.45	38.36	32.69	51.48
Female/male ratio	1.01	1.01	0.68	1.22
Non-Swiss citizens^a^	0.15	0.14	0.01	0.52
No. of physicians	6.45	3	1	510
Physician/10000 inhabitant	7.98	7.29	2.04	38.3
Consultations per inhabitant	3.00	3.00	0.70	7.68
Localization index	56.5%	55.3%	28.9%	97.1%
Market share index	60.0%	60.8%	7.4%	95.3%

^a^Proportion of non-Swiss citizens among residents

The statistical procedures yielded significant effects for all main explanatory variables except for the proportion of non-Swiss citizens (Table 3). The model explained 36.4% of the variation (pseudo-R2 statistics[8]) of the annual per capita consultation rates. The regional GP-density per 10'000 inhabitants was positively and significantly associated with the annual consultation rates in GP practices, and increased by a factor of 0.10 for each additional GP in a population of 10'000 inhabitants (Table 3). The density of specialists in the same region was negatively associated with the frequency of GP-consultations; i.e., the GP-consultation rate decreased by a value of 0.01 for each additional specialist in the population. A similarly significant and "protective" effect was seen for the availability of ambulatory services provided by hospitals (Table 4). Residents in regions having ambulatory hospital departments consulted their GP's 2.81 times per year, whereas residents in "no hospital" regions had 2.99 visits. Higher average age and the proportion of females in the population were both positively and significantly associated with GP-consultations, although no significant effect was seen for the proportion of non-Swiss residents. However, the interaction term between the female-to-male ratio and the proportion of non-Swiss citizens was significant and indicated high consultation rates in communities with a high proportion of females and non-Swiss citizens.

**Table 3:** Parameter estimates of continuous explanatory variables for annual per capita consultation rates with primary care physicians

	Nonstratified, full model		Estimates of language-stratified models		
Variable	Estimate	95% CI	German	French	Italian
Intercept	3.13*	2.75–3.51	3.09*	2.01*	3.14*
Number of GP's/10'000 inhabitants	0.10*	0.01–0.19	0.06*	0.02	0.02
Number of specialists/10'000 inhabitants	-0.01*	-0.02 – -0.004	-0.01*	< -0.01	-0.01
Average age	0.07*	0.05–0.09	0.08*	0.05*	0.10*
Female to male ratio	0.57*	0.08–1.06	0.60	0.94*	0.94
Non-Swiss citizens	-0.27	-3.97-3.43	2.08*	0.59	0.84
Interaction (Female to male ratio * Non-Swiss citizens)	9.39*	4.08–14.71	14.75*	11.73*	8.90

*significant parameter estimates (p < 0.05)

**Table 4:** Least square means (LSM) of annual per capita consultation rates with primary care physicians across language regions, community types, and hospital regions

Variable	Level	LSM	95% confidence limits
Language region	Italian	2.97	2.91–3.04
	Romansh	2.09	1.99–2.19
	Swiss German	3.44	3.26–3.62
	French	3.08	2.74–3.43

Community type	Urban centres	3.01	2.80–3.23
	Suburban	2.98	2.85–3.10
	High income	2.78	2.58–2.98
	Periurban	2.85	2.73–2.97
	Tourist	2.90	2.76–3.05
	Industrial-tertiary	3.14	3.02–3.27
	Rural-commuter	2.87	2.76–2.99
	Agricultural-mixed	2.90	2.78–3.01
	Agricultural rural	2.63	2.50–2.77

Hospital^a^	Not present	2.99	2.88–3.09
	Present	2.81	2.69–2.93

^a ^Hospital providing ambulatory services in the same region.

Pair-wise comparisons of means indicated multiple significant differences between language regions. Only the consultation frequencies for German and Italian vs. Romansh were not significantly different. Least square means indicated furthermore that agricultural/rural and high-income communities had the lowest and industrial/tertiary communities had the highest frequencies (Table 4). All other community types were closely scattered around the overall Swiss mean of a 2.95 per capita consultation rate with a primary care provider.

Significant first-order interaction terms indicated that the effects of GP density and the proportion of non-Swiss citizens were not constant across language regions. Therefore, additional specific analyses stratified by language (Tables 3 and 4) were performed. These data indicated that language-specific effect estimates of the association between GP density and consultation rates were lower than the overall Swiss estimate. GP consultation rates increase significantly by 0.06 for each additional GP in a population of 10'000 inhabitants in the Swiss German-speaking population, whereas considerably lower and non-significant effects were observed in French- and Italian-speaking regions (Table 3). Inverse patterns were seen for the association between the GP-consultation rate and the regional density of specialists. Specialists had a negative effect on GP-consultation rates in all regions. However, this effect was seen only in German-speaking regions significantly associated with GP-consultations (Table 3).

The proportion of non-Swiss citizens appeared, in terms of effect size, to be the most important factor among continuous variables at the community-level for the Swiss German population (Table 3). This implies that GP-consultation rates increased significantly with higher proportions of non-Swiss citizens by a factor of 2.08 for each additional percent of non-Swiss citizens in a community. Less prominent and non-significant effects were seen for this factor in other Swiss language regions.

Variance component analyses showed that service areas account for 39.4% of the total variance of GP-consultations and imply considerable clustering of consultation rates within areas (Table 5). Variance component analyses at the the level of the service areas indicate that only 2.9% of the explainable regional variation is accounted for by the regional density of physicians and that 3.1% and 4.4%, respectively, are explained by specialist density and the presence of a hospital providing ambulatory services (Table 5). Language-specific analyses of variance components show, however, that GP density is an important factor in explaining GP consultation rates in Swiss German populations, but not in French and Italian regions. Inversely, in French-speaking regions, specialist density and hospitals accounted for considerable amounts of regional variation. No or no estimable effects were observed in this context for Italian-speaking regions (Table 5).

**Table 5:** Variance components of regional variables

Variance components	Proportion of regional variation			
	Non-stratified model	German	French	Italian
pseudo R^2^	0.364	0.158	0.187	0.166
Region (ICC)^a^	0.394	0.280	0.194	0.277
No. of GP's/10'000 inhabitants	0.029	0.101	-^b^	-
No. of specialists/10'000 inhabitants	0.031	0.011	0.189	< 0.001
Hospital	0.044	0.016	0.302	-

^a ^intraclass correlation coefficient

^b ^negative variance components

## Discussion

There are several theoretical models in health systems research that are used to explain the availability and utilisation of health related resources [12-14]. Most of these models distinguish between supply- and demand-related factors, and this conceptual differentation was adapted, therefore, for the following discussion.

### Regulation of physician's supply

Swiss health care is characterised by a high degree of decentralisation with strong regional and cantonal influence[15] and high cost [4]. Coordinated and effective interventions to maintain and improve cost-efficiency are thus difficult to achieve. However, as an extraordinary measure, federal legislation in the year 2000 gave the 26 cantonal authorities the authority to restrict the number of ambulatory health care providers. This decision was based mainly on an economic rationale regarding the supply hypotheses of practice variation and should have resulted in a more or less complete stop of new accreditations of Swiss primary care providers in the following years. Apparently, these governmental measures provoked just the opposite effect as the number of newly accredited physicians increased [16]! The association between physician density and the intensity of utilisation of health care resources thus remains a relevant factor in the current debate about reducing costs in Swiss health care. In contrast to previous analyses based on cantonal data, we propose the use of health service areas as the unit of analysis of potential determinants of health care utilisation. This approach reduces the effect of patients seeking care outside their residential area. As this paper demonstrates, without this bias a much better representation of the actual utilization and provision of resources in Swiss primary care can be achieved.

### Determinants of utilization of primary care resources

The analysis of per capita consultation rates across language regions confirms well known cultural differences in Switzerland that extend beyond health care [17]; but the differences we observed between the French and other language regions were larger than expected. Other research in Switzerland showed that these differences are neither related to variations in health status nor to socioeconomic attributes of the respective populations and, furthermore, the same research also indicated a lack of theoretical specification and empirical models in this context [18]. We therefore can only assume that patients in French-speaking communities directly seek care more often from specialists without first consulting a primary care provider. Similar mechanisms may also be at least partially responsible for the observed disparities between the different community types. However, traditionally anchored behavioural patterns and effects of the political and institutional environment including different accessibility of resources can additionally influence the consultation pattern of the underlying populations.

Additional research aimed at these topics is thus needed to investigate the distribution of population-based consultation patterns across language regions, community types, medical specialties, and the corresponding implications on cost for ambulatory care. Presently, we also see strong political pressure to implement managed care, e.g., by establishing physician networks based on gate-keeping. Our results may therefore help guide future developments by setting population-based and regional priorities.

The data provide estimates of the importance of socio-demographic factors relevant for the utilisation of primary care resources in Switzerland. Effects of age and gender confirm well known phenomena in this context. However, particularly in the Swiss German population, the effect of non-Swiss residents far exceeds corresponding effects of other socio-demographic and supply-related factors. These results thus confirm other research in this field, showing a heterogeneity of emigrants across Swiss language regions in terms of gender, ethnical background, migration history and legal status of residency [19,20]. Possible explanations of these findings may be provided within the conceptual framework of cultural epidemiology where health and illness are understood as socio-cultural categories that influence the perception of well being [20]. The findings support, however, ongoing efforts facilitating information, prevention and health care for migrants and improving the cultural competence of care providers [19]. In addition, the findings may also be relevant to many other health care systems facing worldwide refugee and migration phenomena.

Our data provide evidence that the number of physicians accredited in a specific region is an important determinant for the utilisation of Swiss primary care resources. However, the associated effects are not consistent across Swiss language regions and show an inverse relationship between primary care providers and specialist care. The findings not only imply considerable competition for patients between specialists and primary care physicians in the Swiss German regions, but also indicate that the utilisation of primary care resources in these regions is obviously more supplier-driven than in other regions. In contrast, effects of hospital-based supply of ambulatory services remain constant across language regions and indicate consistent patterns of competition: the presence of ambulatory hospitals reduces GP consultations by 0.18 per year and inhabitant.

The question remains, therefore, what are the major driving forces for these findings? Is it supplier-induced demand, clinical uncertainty, or patient needs; and what are the roles of the 'zero risk society' or of medical consumerism? [21] The length of the observation interval may exclude simple fluctuating levels. It may be argued that the available data provide no insight into the decision-making process of individuals seeking and providing care, and consequently that the data do not permit a distinction between demand manipulation and clinical ambiguity [22]. However, our data cover the entire range of non-accident-related primary care reimbursed by social health insurance, including non-clinical incentives and competition factors. These factors together may influence professional activities of physicians working on a fee-for-service basis. Nevertheless, supply-induced demand cannot be excluded as an explanation of our findings, particularly in Swiss German regions. However, variance component analysis reflected not only the decentralized structure of Swiss health care but also provided an estimate of the potential importance of regulatory interventions. From a national perspective, supply-related components contribute only marginally to regional variation in the use of primary care resources. Therefore, the impact of a regulation of supply by, for example, freezing the number of primary care physicians may have only a very limited or even immeasurable effect on the associated resource utilisation. However, language-stratified analyses indicated that considerable amounts of variance are accounted for by the GP-density in Swiss German regions, and specialist and hospital supply in French-speaking regions.

### Limitations and strengths

The results were derived from the claims database of all Swiss health insurers and provide complete coverage of all ambulatory or outpatient services reimbursed by compulsory Swiss social health insurance in 2004. The spatial model used in this study yielded service areas in which, on average, 57% of the population used local health resources, and/or physicians treated local patients in 61% of all consultations. We therefore consider this approach an appropriate spatial representation of utilisation and provision of primary care in Switzerland. It can be debated, however, whether a proportion of 36.4% of explained variance of the outcome is enough to draw relevant conclusions about the effect of physician density and socioeconomic determinants on per capita consultation rates. Our model was based on a set of only nine explanatory variables and on an outcome with known high random error. We consider it unlikely, given the type and extent of data available, that the results are biased due to measurement error. Furthermore, the explanatory variables were derived from aggregated data at the community and service area levels, and not from individuals – the interpretation of the results is thus limited to these two distinct levels of aggregation and cannot be extended for individual patients or physicians. Given these factors, we believe the achieved amount of explained variance merits publication of the data. Furthermore, the distinct multi-cultural population mix in Switzerland offers excellent conditions for studying health care use across different cultures. A potentially significant imitation in interpreting our data on resource use and volume in Swiss primary care is related to the fact that the referral data are still incomplete. A further limitation derives from the fact that the activity levels of the physicians were not accounted for in the analysis, i.e., full-time and part-time physicians were equally weighted. Other limitations attach to the cross-sectional nature of the study, since service areas are not stable constructs over time because the availability and utilisation of resources may change rapidly. The methods applied in this work are not perfect, but they may be helpful in describing, understanding, and managing the underlying processes.

## Conclusion

Our study confirms other reports documenting large small-area variations in the utilisation and provision of health care resources in the multicultural population of Switzerland [15,23]. In contrast to other research, we propose an alternative spatial model for analysing resource utilisation in order to provide comparable, utilisation-based service areas. We quantified the effects of health system factors, including physician density, as key elements of per capita consultation rates in Swiss primary care. Physician density appeared to be a relevant factor associated with resource utilisation. The effects of primary care providers and specialists were, however, different across language regions, and hence, cultural background. Only limiting the number of care providers may not bring significant changes in volume. Therefore, additional measures to reduce unexplained variation in volume, such as providing physicians with data on their resource use compared with clinical reasoning based on evidence-based guidelines and the practice patterns of their peers, may be evaluated further. Early experiments using educational forms of feedback proved successful in changing physician behaviour and the associated costs in the long run [24,25].

## Competing interests

The author(s) declare that they have no competing interests.

## Authors' contributions

AB obtained the mandate to perform the study, he performed all statistical analyses and wrote the first draft of the manuscript, BK reviewed and completed the manuscript with reference to all aspects of primary care.

## Pre-publication history

The pre-publication history for this paper can be accessed here:


